# Quantification of Overnight Movement of Birch (*Betula pendula*) Branches and Foliage with Short Interval Terrestrial Laser Scanning

**DOI:** 10.3389/fpls.2016.00222

**Published:** 2016-02-29

**Authors:** Eetu Puttonen, Christian Briese, Gottfried Mandlburger, Martin Wieser, Martin Pfennigbauer, András Zlinszky, Norbert Pfeifer

**Affiliations:** ^1^Department of Remote Sensing and Photogrammetry, Finnish Geospatial Research Institute, National Land Survey of FinlandMasala, Finland; ^2^Department of Remote Sensing and Photogrammetry, Centre of Excellence in Laser Scanning Research, National Land Survey of FinlandMasala, Finland; ^3^Department of Geodesy and Geoinformation, Technische Universität WienVienna, Austria; ^4^EODC Earth Observation Data Centre for Water Resources MonitoringVienna, Austria; ^5^RIEGL Laser Measurement SystemsHorn, Austria; ^6^Balaton Limnological Institute, Centre for Ecological Research, Hungarian Academy of SciencesTihany, Hungary

**Keywords:** terrestrial laser scanning, plant movement, chronobiology, circadian rhythm, time series

## Abstract

The goal of the study was to determine circadian movements of silver birch (*Petula Bendula*) branches and foliage detected with terrestrial laser scanning (TLS). The study consisted of two geographically separate experiments conducted in Finland and in Austria. Both experiments were carried out at the same time of the year and under similar outdoor conditions. Experiments consisted of 14 (Finland) and 77 (Austria) individual laser scans taken between sunset and sunrise. The resulting point clouds were used in creating a time series of branch movements. In the Finnish data, the vertical movement of the whole tree crown was monitored due to low volumetric point density. In the Austrian data, movements of manually selected representative points on branches were monitored. The movements were monitored from dusk until morning hours in order to avoid daytime wind effects. The results indicated that height deciles of the Finnish birch crown had vertical movements between -10.0 and 5.0 cm compared to the situation at sunset. In the Austrian data, the maximum detected representative point movement was 10.0 cm. The temporal development of the movements followed a highly similar pattern in both experiments, with the maximum movements occurring about an hour and a half before (Austria) or around (Finland) sunrise. The results demonstrate the potential of terrestrial laser scanning measurements in support of chronobiology.

## Introduction

Terrestrial laser scanners have gone through rapid development during the past 10 years (Dassot et al., 2011). They produce accurate 3D point clouds of target objects often down to millimeter resolution. Thus, the point clouds provide detailed information about the structure and spatial properties of the targets.

In addition to structural modeling and scene mapping, terrestrial laser scanning (TLS) data are widely used in engineering applications to monitor possible changes in the targeted objects or in a target area. TLS-based change detection studies cover a wide range of different applications. These include geodynamic processes such as landslide detection and monitoring (Travelletti et al., 2008; Ghuffar et al., 2013) and morphodynamic changes in coastal beaches (Lindenbergh et al., 2011), thermal karst formations (Barnhart and Crosby, 2013), or in riversides (Milan et al., 2007; Vaaja et al., 2011; Saarinen et al., 2013). Short interval scans are also used in determining dynamic structural stress (Grosse-Schwiep et al., 2013). TLS techniques are utilized to improve safety in quarries and mines by monitoring wall stability over time (Abellan et al., 2011; Hu, 2013; Kovanic and Blištan, 2014). In vegetation studies, TLS is being actively used in static forest and forest parameter mapping (Hopkinson et al., 2004; Moskal and Zheng, 2012; Liang, 2013), tree modeling (Fleck et al., 2004; Hosoi and Omasa, 2006; Bucksch and Fleck, 2011; Eysn et al., 2013; Raumonen et al., 2013), and in estimating forest biomass (e.g., Kaasalainen et al., 2014).

Although TLS is utilized in a wide range of both temporal and vegetation studies for scientific and engineering applications, one field of study which has not yet gained wider interest in the TLS community is circadian or diurnal and nocturnal changes in vegetation. In ecology and plant physiology, these changes in plants and their driving factors have been studied intensively for a long time. It has been known for centuries that plants show diurnal cycles of leaf motion, described as “sleep” already by Darwin and Darwin (1880). It has been observed that these motions also happen if the plant is placed in darkness, therefore suggesting an internal mechanism for measuring time. The molecular mechanism of this circadian oscillator has been most extensively studied in *Arabidopsis* (Barak et al., 2000), but orthologues of the *Arabidopsis* genes controlling the diurnal rhythm of flowering time have been identified in various tree species such as Poplar (*Populus tremula*) and Chestnut (*Castanea sativa*) (Solomon et al., 2010). However, for obvious reasons, carrying out controlled experiments or even quantitative observations on fully grown trees is much more difficult, therefore both the background and the effects of sleep motions in trees are less well-understood. Circadian rhythms of leaf motion are evident for some tree species such as *Robinia pseudoacaia*. The movement of leaves is connected to changes in turgor pressure (Holmes and Shim, 1968) which is controlled by the osmotic state of the cells. Changes in water transport and in the concentration of various metabolites result in changes in osmotic flow and thus, through changes in the shape of individual plant cells, eventual movement at the scale of individual plants or plant parts. The literature identifies two common reasons that drive these changes, namely plant water balance (Chapin et al., 2002) and photoperiodism (McClung, 2006; Nozue and Maloof, 2006; Sysoeva et al., 2010). It would be plausible that the changes in turgor pressure resulting in a circadian rhythm of leaf movement also apply to the branches and thus cause a circadian rhythm of branch movements in trees. However, in an earlier case study where quantification of diurnal movement was attempted using height measurement of weighted lines attached to branches in a Walnut orchard (*Juglans regia*), diurnal change was found insignificant (Way et al., 1991). To our best knowledge, circadian movement of tree branches has never been successfully quantified before in the presented level.

Methods to monitor plant water balance at a diurnal time scale include: (a) leaf or fruit sample collection and water content measurements (Klepper, 1968; Acevedo et al., 1979); (b) sap flow monitoring (Köstner et al., 1998); (c) leaf and stem conductance measurements (García-Orellana et al., 2013); (d) leaf and stem water potential, photosynthetic capability and hydraulic conductivity measurements (Andrews et al., 2012); (e) branch growth and xylem morphometry measurements (Correia and Martins-Loucao, 1995; Correia et al., 2001). Most of these are invasive processes that involve sampling and are difficult (but possible) to carry out regularly in intervals of a few hours (Chapotin et al., 2006). Therefore, in case of trees, the most common way of observing circadian rhythms is with a dendrograph, an instrument that measures changes in tree diameter or circumference with sub-millimeter precision (e.g., Pesonen et al., 2004).

In order to monitor photoperiodism, the amount of light received by plants can be controlled by constructing external shading structures (Wayne and Bazzaz, 1993) or by using external lighting setups with selected filters (e.g., Mockler et al., 2003). Alternatively, in order to monitor the internal clock, a plant can be placed in continuously lit or dark conditions to observe periodic changes in its physiology.

Plant physiology measurements are localized and typically consider selected parts of a plant. The measurements often take place in laboratory conditions. This presents a clear challenge when results are extrapolated to model wider areas of multiple plants. Acquiring results also involves a significant amount of manual labor, as experiment setups and sample collection are hard to automatize.

TLS measurements offer a potential solution to generalize plant physiology results on larger spatial scales, like whole individual trees, or on orchard plot, and stand levels. Laser scanners can measure individual targets accurately tens of meters away with sub-centimeter point resolution. Moreover, scanning can be performed outdoors with short intervals between individual scans. Furthermore, as the scanners are active measurement devices that both produce and receive the signal, they are insensitive to varying external lighting conditions, i.e., available sunlight and cloudiness.

Laser scanning point clouds cannot provide direct biochemical parameters from plants, but they can be used in plant shape and dimension monitoring over time (e.g., branch inclination, branch, and stem swelling, leaf inclination distribution at crown level). If a clear correlation between the geometric changes in point clouds and laboratory or *in situ* results can be established and verified, the parameterized spatial changes can then act as proxies that estimate physiological changes in a plant.

Puttonen et al. (2015) detected and reported birch branch movements during a day-long classification study. The study was carried out with the Finnish Geospatial Research Institute (FGI) Hyperspectral Laser Scanner (HSL) (Hakala et al., 2012). The movements were detected from the variation of the birch Normalized Difference Vegetation Index (NDVI) response during on an observation period of ca. 26 h. A more detailed inspection revealed a visible change in birch branch stances over time. The study is to our knowledge the first to report spatial changes in tree branch geometry over a day-long cycle. However, the authors did not attempt to quantify the movement amplitude.

Hitherto, vegetation time series in TLS have been collected mainly for longer scale time series analyses, typically to determine seasonal changes in tree canopy (e.g., Hosoi and Omasa, 2009; Nevalainen et al., 2014; Hakala et al., 2014; Portillo-Quintero et al., 2014; Calders et al., 2015; Griebel et al., 2015).

In imaging, longer term time-series studies have been carried out both in the field and in a laboratory with a close range setup (e.g., more recently by Li et al., 2013; Nijland et al., 2014). However, the use of cameras inherently limits the experimental setup to daylight hours or requires the use of external light sources. Additionally, radiometry measurements and their calibration are typically rather complex. Furthermore, even a short use of external light sources may interrupt the plant photoperiod (e.g., Salisbury, 1981). Meanwhile, since laser scanners are active measurement systems their working efficiency is stable in nighttime conditions with no external light (Arslan and Kalkan, 2013). With modern laser scanners, the laser beam footprint up to a few centimeters in diameter illuminates only a localized patch of the plant surface at a time. Moreover, the footprint swipes over each spot in sub-second scale and the typically used infrared (IR) wavelengths have strong reflectance from green vegetation. Therefore, most of the transmitted energy is not absorbed by the plant. Thus, we assume that plant disturbance with a modern laser scanning system is not significant during an individual scanning measurement.

This study now aims (i) to analyze to which degree overnight birch branch movements can be measured with two different TLS scanners and (ii) whether quantifications of crown/branch movements are possible. With these goals in mind, we aim to show that TLS data provides an effective tool to detect and to monitor circadian changes in tree geometry with a centimeter scale spatial and within-hour temporal resolution. As the main focus of the study is in confirmation and quantification of geometric changes in birch branches over time, without further investigation of the cause or mechanism of these movements, the measurement setups did not include comprehensive weather or soil data.

## Measurements and data

This section is structured as follows: In section Measurement Sites and the Collected Data, descriptions of both the Finnish and the Austrian measurement sites and data collected from them are given. Section Object Point Cloud Delineation describes object delineation from the scanned point clouds. Section FGI Hyperspectral Lidar provides a detailed description of FGI HSL and its properties. Section RIEGL VZ-4000 Long Range Laser Scanner gives a detailed description of the RIEGL VZ-4000 laser scanner.

### Measurement sites and the collected data

Measurements were carried out in two geographically different locations, in southern Finland (Kirkkonummi, 60°09′40″N, 24°32′48″W) and in northern Austria (Horn, 48°39′31″N, 15°39′48″E). Both measurements were performed in the middle of September close to the solar equinox to guarantee approximately similar lengths of night at both sites (http://www.timeanddate.com, accessed on 25th February 2015). Measurement dates were 13–14 September 2013 (Finland) and 19–20 September 2014 (Austria). In both measurements, the test trees were measured from sunset to sunrise. In Finland, the nighttime measurements lasted about 11 h in total, during which 14 separate scans were collected with FGI HSL. Scan intervals were approximately 1 h. Near sunrise and sunset the scan interval was shortened to 40 min. In Austria, data acquisitions were repeated every 10 min for about 12 and half hours, resulting in 77 separate scans.

**Figure 1:**
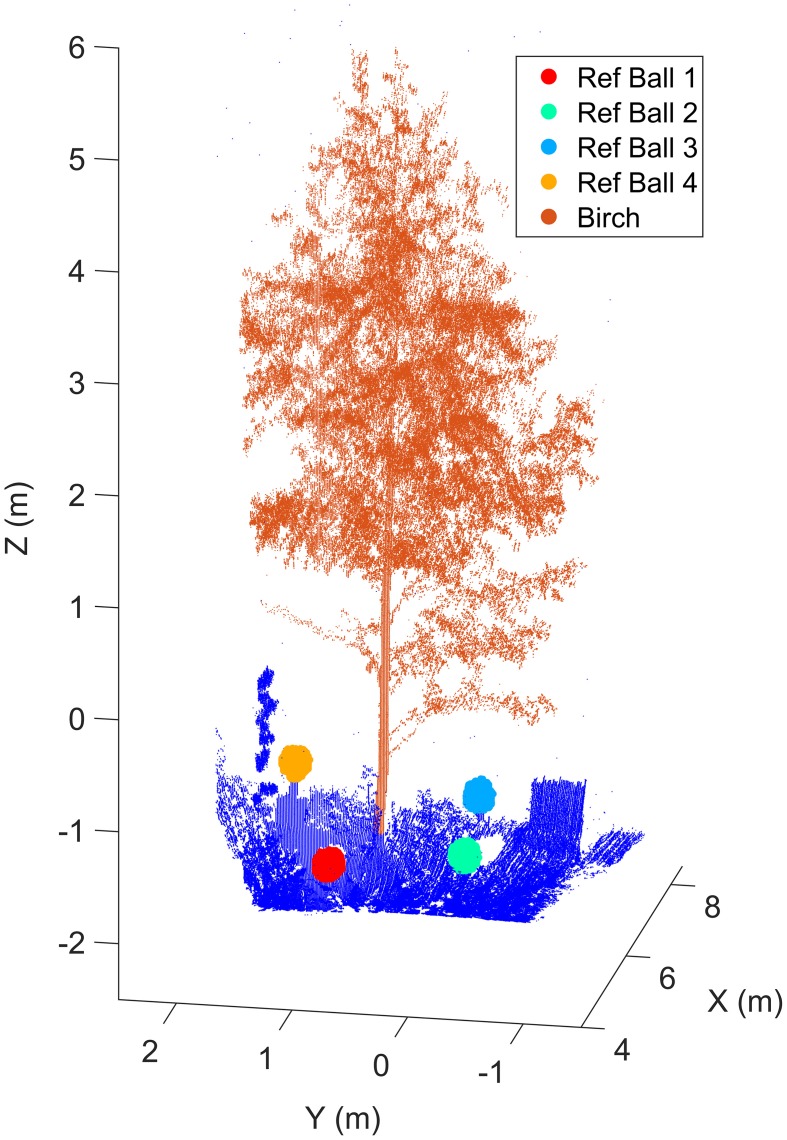
**The birch and reference markers at the Finnish measurement site**. Reference markers were used in monitoring the HSL ranging stability. Reference target sizes in the figure have been emphasized for visualization purposes. The scanner was located in the origin (0,0,0).

The Finnish measurement site was located on a shallow slope facing southward. The size of the test site was about 7 × 20 m^2^. The site included the main target, a small silver birch (*Betula pendula*). Low understory, a large silver birch and goat willows (*Salix caprea*) were located behind the target. The site was partially surrounded from its eastern side with a sparse, half-open canopy of full-grown birches. The nearby FGI building was located on the western side of the site. The shadow of the building shaded the target site for about half an hour before the sunset. Figure 1 illustrates the Finnish test tree and the reference markers placed around it. The markers were used to validate the distance measurement stability over the experiment. The measurement setting also included an external reference plate (Spectralon®) to calibrate laser radiometry during the experiment. The FGI HSL was mounted on a solid platform and a plastic tent was set up around the scanner to protect it from possible rain and moisture. A heating fan was kept on whole night time inside the tent. This guaranteed a constant airflow and prevented possible surface condensation.

**Figure 2:**
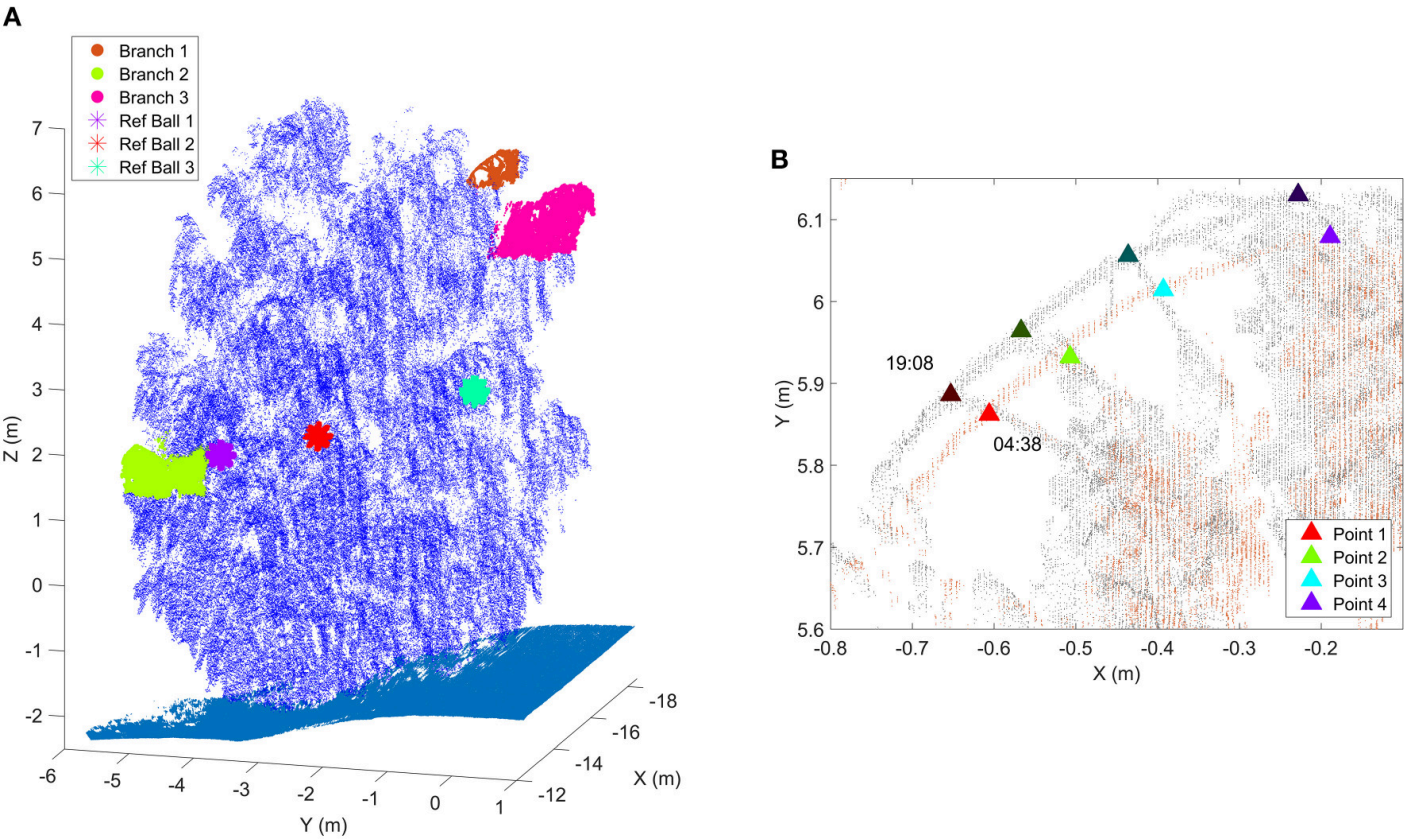
**(A)** The birch at the Austrian test site. The selected target branches and the three detected reference markers have specific coloring. Ground points are presented with cyan. Reference target sizes have been emphasized in the figure for visualization purposes. The scanner was located in the origin (0,0,0). **(B)** A close-up of Branch 1 at two different times. Triangles represent the manually selected representative points of which total 3D movements were followed over time. Dark triangles represent the initial point locations at sunset (19:08), bright triangles the corresponding point locations at the time of the movement maximum (04:38).

The Austrian measurement site was located on the northern part of an outdoor test and calibration range, an open, cut grass field of size about 130 m by 100 m. The target birch had four stems and it was standing about 10 m away of a manufacturing hall. Thus, the tree was not shaded during sunrise or sunset. The scans were taken to the westward direction and the laser scanner was under a protective roof. Four white polystyrene foam spheres were attached to the birch as reference markers to detect branch movement. Figure 2 illustrates the Austrian test tree and the branches of which movements were followed during the experiment. The branch point clouds were manually selected in CloudCompare software [Available in: http://www.cloudcompare.org/ (Girardeau-Montaut, 2014)].

The weather conditions in both measurement sites were similar during data acquisition. The air was calm, with no wind (qualitative observation), during the night. For this reason, the time of sunset was selected as the initial point for monitoring the branch movement. There was no rainfall during either measurement. In the Finnish test site, the leaves of lower branches were verified not to have visible moisture condensation on their surfaces during nighttime.

### Object point cloud delineation

A manual workflow was used to delineate birch and reference target point clouds spatially from the whole point cloud. The same procedure was used for both datasets.

The differentiation was started by viewing an object (birch or reference target) point cloud as a 2D projection from a user selected angle and then cutting the object outline by hand with a clear buffer zone. Points inside the outline were included and the rest were rejected. After the cut, a new 2D projection of the included points was taken from another angle and the manual cutting was repeated. In total, 4–5 projections were required to form a sufficiently accurate 3D delineation for the objects in both cases. As the objects had no spatial overlap in either measurement setup, there was no mixing between the object point clouds. The buffer zones in each cut were selected so wide that possible temporal movements within the object point cloud and noise point fluctuations around the object edges (e.g., due to partial hits) were captured for all measurements. All points within the selection area were kept. Intensity-based filtering was not performed.

### FGI hyperspectral lidar

The FGI Hyperspectral Lidar (HSL) is a laser scanning system that transmits hyperspectral (white) laser pulses with a continuous spectrum of 400–2500 nm to the target. It can measure up to eight separate wavelength bands from returning pulses. The number of bands is limited by the spectral sensitivity of the silicon detector, but the wavelengths are selectable within the transmittance range. In this study, spectral information was used only to emphasize differences between leaf and stem returns with the Normalized Difference Vegetation Index (NDVI).

The main components of the HSL system are the SM5-he supercontinuum laser source (Leukos, Limoges, France), a 2D scanning mechanism (Newport Corp., Irvine, CA, USA), the wavelength separating spectrograph (Specim, Oulu, Finland), a 16-channel high speed detector element (First Sensor AG, Berlin, Germany), and the measurement computer with digitizer cards (National Instruments Corp., Austin, Texas, USA). The HSL works by sending laser pulses to the target in a sweeping pattern and then recording returning waveforms for each wavelength band detected. The waveforms are digitized with 1 GHz frequency, thus giving the system a nominal 15 cm range resolution. However, individual laser pulses can be localized to the waveforms with a *de facto* range resolution of a few centimetres. The HSL can measure up to 5000 waveforms per second and a maximum of three discrete returns are fitted in each waveform.

A more detailed description of the HSL system, its properties, and its measurement configuration are given in articles by Hakala et al. (2012) and Nevalainen et al. (2014).

The properties of both the FGI HSL and RIEGL VZ-4000 scanners are compared in Table 1.

**Table 1:** **Property comparison between the laser scanners used in the Finnish and Austrian test sites**.

**Laser system**	**FGI HSL LEUKOS-SM-X-OEM**	**RIEGLVZ-4000**
Laser wavelength (nm)	420–2100	1550
Average output power (mW)	41	Setting dependent
Measurement rate (kHz)	5.3	23–222
Pulse width	≤ 1 ns	3 ns
Central wavelength of a detected channel (nm)	545.5, 641.2, 675.0, 711.0, 741.5, 778.4, 978.0	1550
Channel FWHM (nm)	20	–
Collecting optics field of view	0.2°	–
Transmit beam divergence	0.02°	0.009°
Transmit beam diameter (cm) at stem distance (*m*)	0.7 (*7.5*)	1.9 (*14.7*)
Scanning resolution, horizontal	0.1°	0.002°
Scanning resolution, vertical	0.02°	0.002°

### RIEGL VZ-4000 long range laser scanner

The RIEGL VZ-4000 has been developed for surveying applications in which very long ranges are required (RIEGL, 2014). The scanner has a net measurement rate of up to 222 kHz. The maximum measurement range of the VZ-4000 is 4 km for targets with 90% reflectance at 23 kHz measurement rate. At 222 kHz the instrument is still capable of measuring targets with only 20% reflectance up to 1000 m.

The RIEGL VZ-4000 works, like the HSL, by sending laser pulses to the target, but analyses the recorded waveform internally. The nominal ranging precision is 10 mm.

The VZ-4000 can be controlled via its built–in touch screen, via WIFI or LAN. It has a built-in camera, GPS receiver, compass, and tilt sensor, and there are interfaces to attach an additional camera (e.g., IR camera) and/or a high-precision RTK GNSS.

## Results

### Finnish point cloud time series

A total of 14 scans were selected from the Finnish point cloud time series in order to determine the temporal variation in the birch crown. Since the FGI HSL is a prototype device, its relative ranging precision during the experiment was first validated. Movements of the reference targets set around the birch were monitored for the duration of the experiment. Table 2 lists the reference target distances to the scanner and their relative movements compared to the initial scan. The target ranges and movements were calculated with respect to the target center that was determined with a least squares sphere fitting (MATLAB code by Alan Jennings, available in www.mathworks.com/matlabcentral/fileexchange/34129). The table shows that the reference targets were detected reliably and with higher precision (less than 5 mm standard deviation) than the nominal sampling frequency of the system would imply. This assumption is valid as long as the laser returns can be expected to come from a single reflection, but cannot be directly generalized to more irregular targets, like differently positioned leaves, or to partial hits coming from edges. Nevertheless, the ranging stability test verified the HSL system accuracy for fixed target distances and that the HSL point cloud did not present a systematic drift during the measurement period.

**Table 2:** **The ranging stability of the FGI HSL point cloud during the experiment**.

**Ref Ball No**.	**Distance from the scanner (m)**	**Distance from the scanner, standard deviation (m)**	**Fitted radius, mean (m)**	**Fitted radius, standard deviation (m)**
1	6.540	0.002	0.120	0.001
2	7.113	0.002	0.122	0.001
3	8.517	0.003	0.120	0.002
4	7.784	0.004	0.125	0.002

*The table shows the reference target distances from the scanner and the radii fitted in their point clouds. Both the ranges and radii were compared to the initial measurement close to sunset. All standard deviation values were calculated from 14 scans*.

After the range precision measurements, points were manually delineated into a new individual point cloud of the studied tree for each measurement as described in Section Finnish Point Cloud Time Series. Then, the point cloud was differentiated into crown and stem point clouds. The differentiation was based on the NDVI distribution of all birch points. The differentiation was performed using a hard NDVI threshold of 0.2, where the points with NDVI values below the threshold were classified as stem and thick branches. The bounding box of the crown point clouds had dimensions of 3.7 × 3.0 × 6.6 m^3^ (depth, width, height) when averaged over all measurements. The median point number for the crown point clouds was 154,310 ± 10,030 points corresponding to 6.5% variability in the total point number.

**Figure 3:**
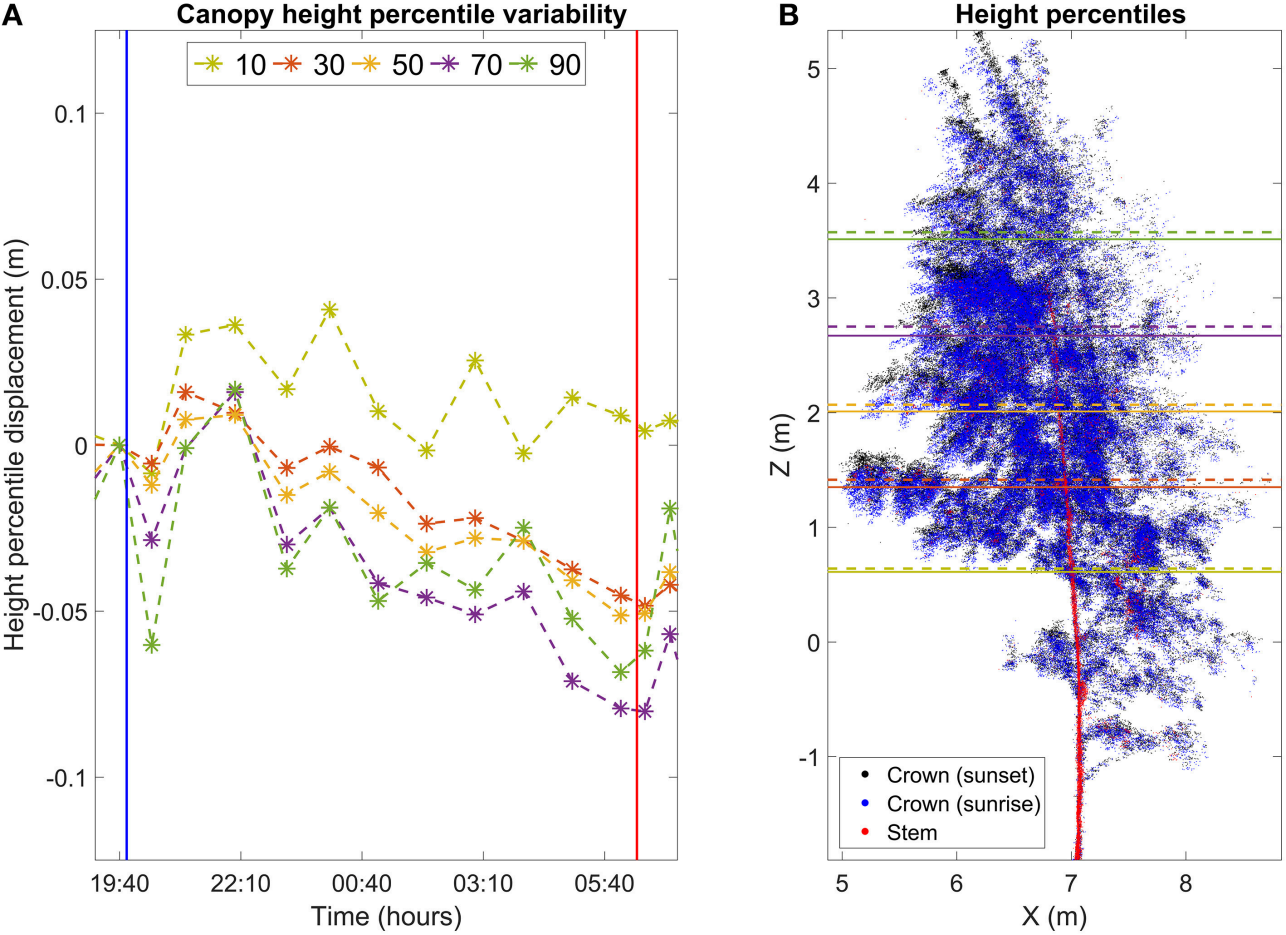
**(A)** Movement of height percentiles of birch crown points in the Finnish dataset. Height and time are reported with respect to the scan closest to sunset. Vertical lines mark sunset (blue) and sunrise (red). **(B)** Maximum movement of height percentile positions overnight. Black points represent the birch crown at sunset. Blue points represent the birch crown at the time of sunrise. Red points represent the birch stem and the thickest branches. The stem and crown points were differentiated with NDVI thresholding. Horizontal lines represent the height percentile locations at sunset (dashed, about 19:40 o'clock) and in morning (solid, 06:40 o'clock).

The goal of the division was to select the returns reflecting from leaves and most of the branches of the birch and to leave the trunk and the thickest branches out of the analysis. After this, five different height percentiles were calculated for the crown point cloud for each scan (Figure 3). The height percentiles in the analysis were: 10th, 30th, 50th, 70th, and the 90th percentile. For example, the 90th percentile is located at the height at which 90% of the points of the whole cloud are below it. Height percentiles were calculated because a manual selection of corresponding points in consecutive scans was not possible due to the low volumetric point density. Height percentiles, on the other hand, are robust descriptions of the measurement height distribution and should therefore allow a reliable tracking of vertical movements of the crown point cloud.

**Figure 4:**
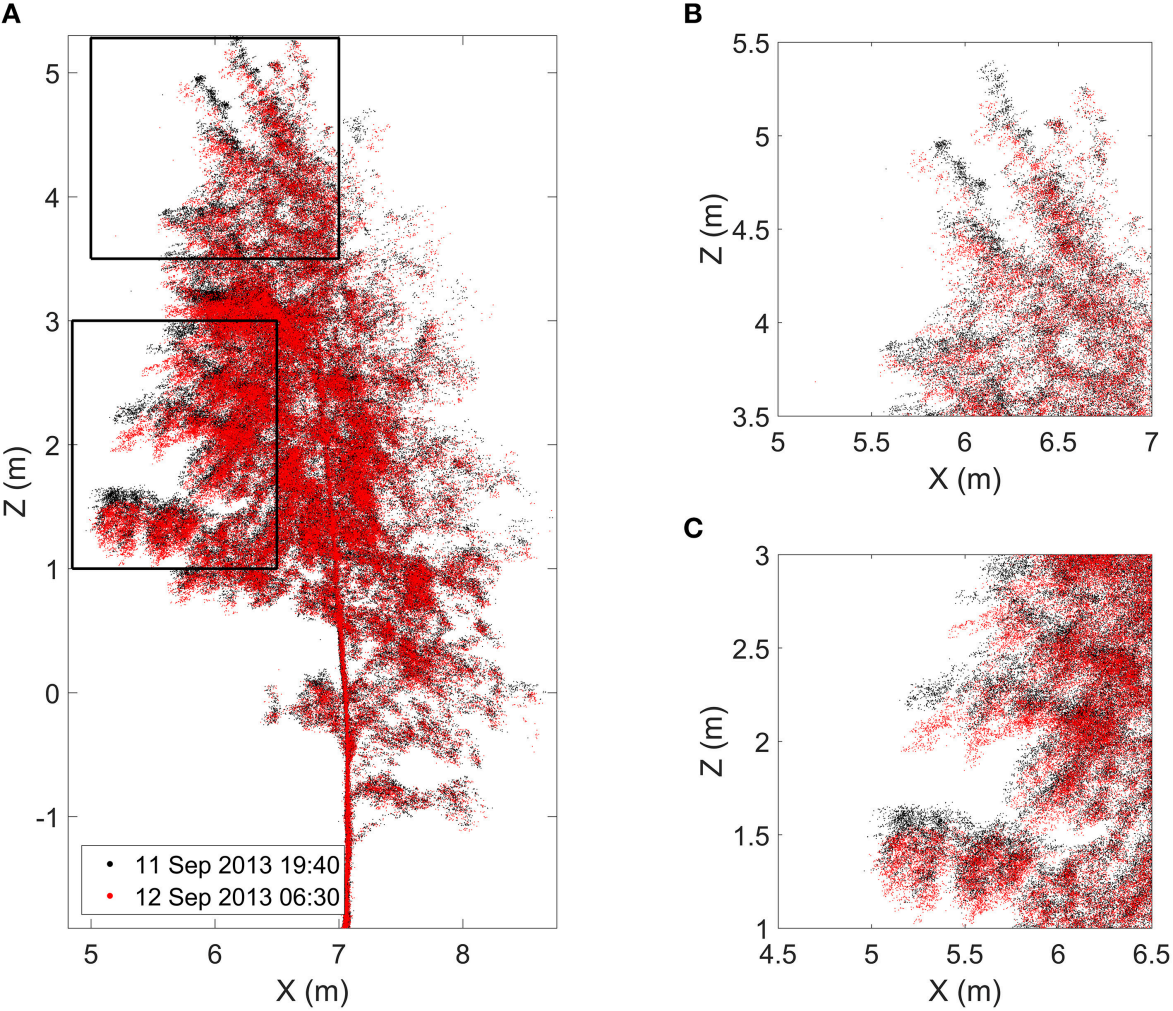
**(A)** Finnish birch point cloud profiles at the time of sunset (black) and at the time of the maximum movement around 06:40 o'clock (red). Black frames mark zoomed in boxes in the upper **(B)** and in the lower **(C)** crown. Video 1 visualizes the geometrical change in the Finnish birch point cloud over night. It is provided in the Supplementary Material.

The results show clear and systematic temporal variation in height statistics of the birch crown point cloud (Figures 3, 4, Video 1). In Figure 3, all height percentiles show a declining temporal trend about 1 h after sunset (blue vertical line). The four highest percentiles also share another common trend: the height percentiles after sunrise (red line) were still lower than their sunset value. At this point, their values have decreased about 0.05 m from their sunset value. After reaching the minimum, all four percentiles show a rapid return toward the sunset value. The return takes about 3 h. The lowest percentile shows a differing temporal response compared to the others. Its value declines more slowly than the others during night, to about 0.03 m from the sunset value. Furthermore, the lowest point is reached about an hour later than for the other percentiles. The overall trend of the lowest percentile is also not as clear as with the other percentiles: the declining trend is less clear and shows more variance.

### Austrian point cloud time series

The RIEGL VZ-4000 was factory calibrated and mounted on a solid pillar throughout the experiment, and so its ranging properties were not inspected as with the Finnish datasets. The bounding box around the birch had average dimensions of 6.0 × 7.0 × 9.3 m^3^ (depth, width, height). The median point number inside the bounding box was 9,388,000 ± 724,000 corresponding to 7.7% variability in the total point number.

**Figure 5:**
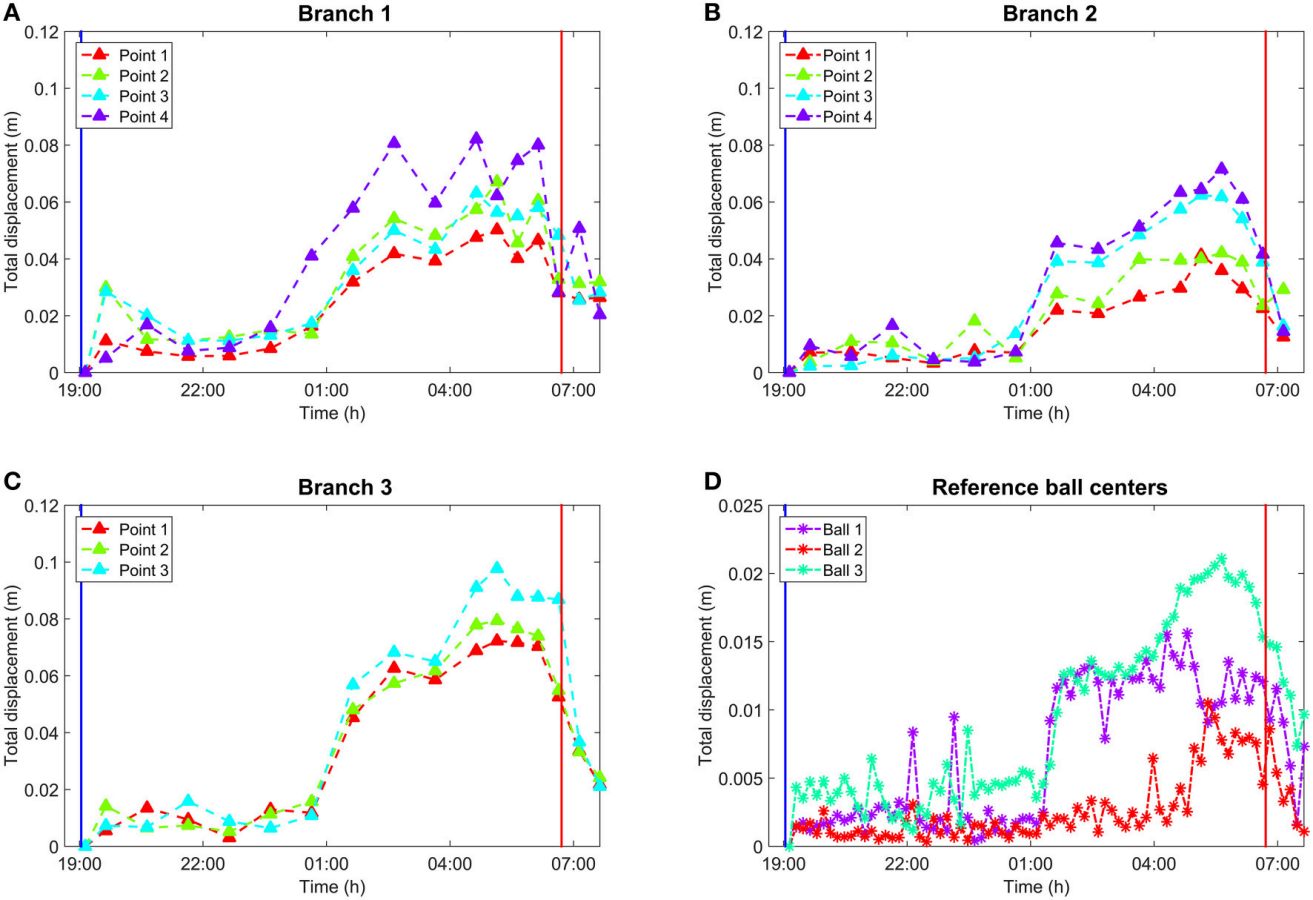
**(A)** Total movements of the representative points on Branch 1 over time. **(B)** Total movements of the representative points on Branch 2 over time. Branch 2 is missing the last epoch due to internal occlusion. **(C)** Total movements of the representative points on Branch 3 over time. **(D)** Total movement of reference target centers over time. Vertical lines in all subplots mark sunset and sunrise. All values in **(A–D)** represent the absolute 3D movement compared to the initial point selection at the scan closest to sunset. Manual point selection in **(A–C)** was performed by picking scans once per hour which was sufficient to represent the general movement trend over time. In **(D)**, reference centers were extracted automatically for all 77 scans. The reference spheres were attached on thicker branches and closer to the stem than Branches 1–3. This resulted in smaller movement amplitudes in the sphere center movement than with the branches.

Manual branch point selection was carried out for three branches. Reference target movement was measured from the fitted sphere center. Three of the four attached reference targets were detected reliably. The fourth one was not detected due to it being occluded from the scan position by birch branches and leaves. Figure 5 illustrates the total movements of the selected points on the branches and the reference target centers.

The graphs in Figure 5 show clearly how all points measured on branches had a similar movement pattern over time. The absolute branch point movement was limited mainly to within 2 cm, with the exception of a few points, until 5 h after the sunset. Thereafter the movement trend began to increase, reaching its maximum around 9–11 h after sunset. The maximum movements varied from 5.0 to 8.5 cm for Branch 1, from 3.5 to 7.0 cm for Branch 2, and from 6.5 to 10.0 cm for Branch 3. After the movement maximum was reached, all branches returned rapidly toward their sunset values around sunrise (about 07:00 h in the graph). The movement was dominated by the downwards component, but included also a comparatively small radial contribution.

In addition to the similar time response, the amplitude of point movements was dependent on the point distance from the trunk in general. For every branch, the point indexing was selected so that the index increased when moving away from the trunk (Figure 2). As a general trend, points with higher index values moved more compared to points with smaller indices. The subplots show exceptions from this rule at different times, but the general trend is visible.

For reference targets attached to the branches, movement of their centers showed a similar, but attenuated, temporal trend compared to the branch points. Reference targets 1 and 3 showed relatively little movement until about 6 h after sunset, excluding occasional noise points. Then, both reference target centers showed a sharp movement with amplitude of 1 cm. The movement then increased until 05:30 o'clock, after which it diminished rapidly by about an hour after sunrise. Reference target 2 differed from the two others in that its movement amplitude was lower and the maximum movement occurred already around 05:00 o'clock. However, the movement diminished in the same way as in the case of the other reference targets.

### Considerations on result comparability

The results show similar temporal behavior between the two birches. This is despite the difference in location of over 12 degrees of latitude, and the difference in form of growth: the Austrian tree was a cultivar with mainly hanging branches while the Finnish study tree had more erect branches with only the branchlets hanging. Growing conditions in the measurement sites also differed from each other, the Finnish site was more covered and in a slope, whereas the Austrian site was on an open and flat grass field.

Although both experiments showed clear temporal correlation in birch branch movement overnight, the absolute branch movements cannot be compared directly. The spatial resolution obtainable with the FGI HSL was not detailed enough to determine individual branch points reliably between consecutive scans. Thus, the temporal development of the Finnish birch was treated on a crown level. The use of crown point height percentiles shows how the different parts of the crown move with respect to each other, but this level of detail was not sufficient to analyze the movement amplitude of individual branches.

The RIEGL VZ-4000 point clouds were dense enough for individual representative point monitoring. In addition, the reference markers attached to birch branches gave another point of reference to determine the movement amplitude. Since the representative points on branches were picked by hand, this resulted in an uncertainty of about 1 cm. However, all manually selected branch points showed systematic movement amplitudes of several centimeters that was several times larger than the point ranging uncertainty and were thus interpreted as a result of changes in branch position. In the Finnish dataset, the thickest lower branches adjacent to the stem were measured to be about 15 mm in diameter. In the Austrian dataset, the selected branches were about 10–20 mm in diameter as measured from the point clouds.

In order to better compare the results for estimating required point densities for future measurement planning, a comparative table of the two measurement setup is presented in Table 3. The table sums up both the differences and the similarities between the measurements.

**Table 3:** **Comparison between the similarities and differences in the Finnish and Austrian measurement setups**.

**Similarities**	
**Type**	**Description**
Tree species	Birch, *Betula pendula*
Time of the year	Finland: 13–14 of September
	Austria: 19–20 of September
Weather conditions	Clear weather with some overcast No observed wind (operator observation) No rainfall No visible surface condensation
**Differences**	
Geographic location	Separation distance: about 1500 km
	Difference in latitude: about 12°
Crown bounding box dimensions (depth × width × height, m^3^, median)	Finland: 3.7 × 3.0 × 6.6
	Austria: 6.0 × 7.0 × 9.3
Total volume of the tree point cloud (sum over populated voxels, m^3^, median)	Finland: 4.20
	Austria: 45.23
Growing spot	Finland: Semi-open, on a slope
	Austria: Open, flat field
Laser scanning systems	Finland: Experimental (FGI HSL)
	Austria: Commercial (RIEGL VZ-4000)
Year of measurement	Finland: 2013
	Austria: 2014

**Figure 6:**
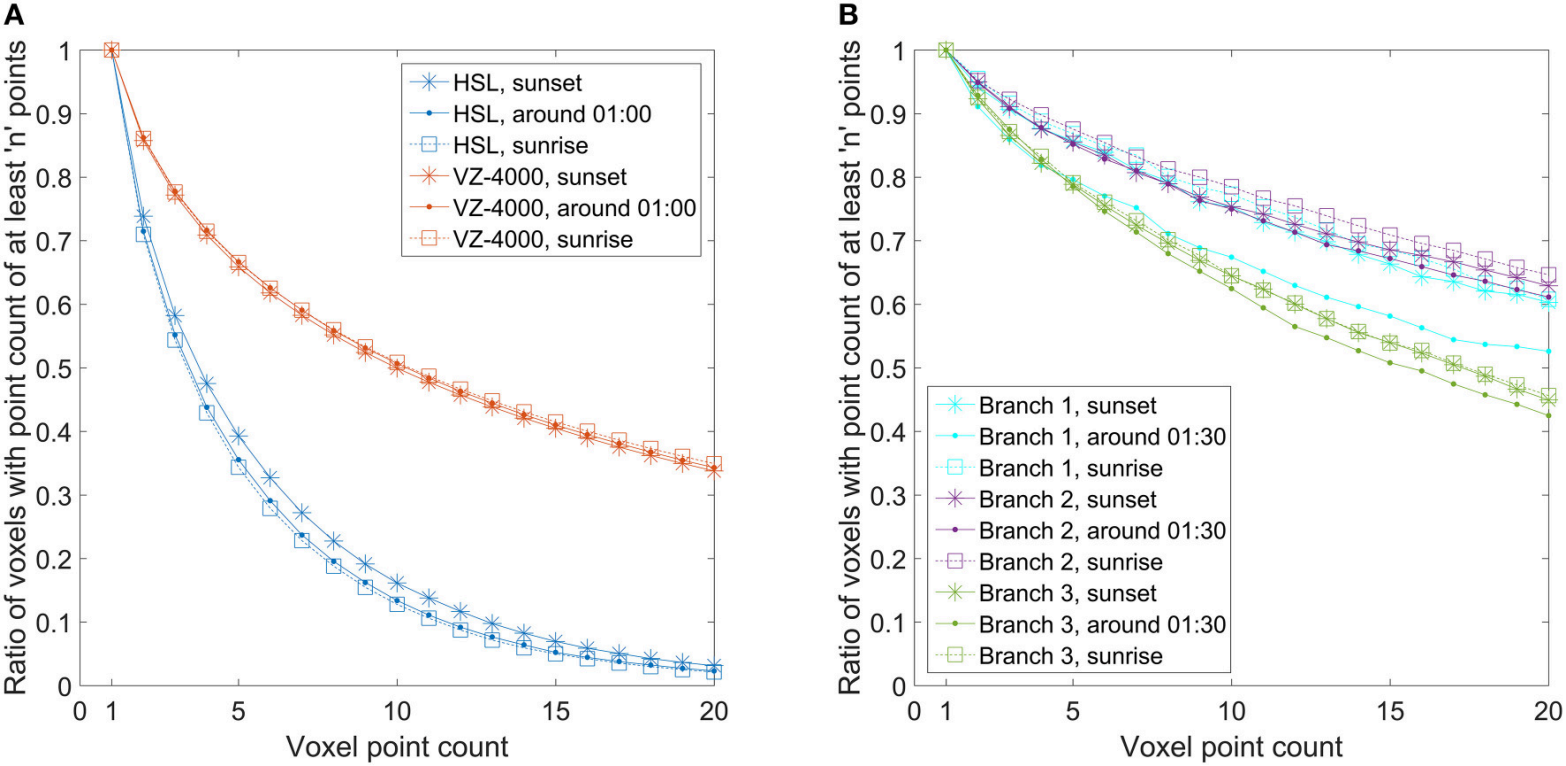
**Normalized cumulative number of voxels populated with at least N points**. Voxel size was set to 5 × 5 × 5 cm^3^ in each Finnish and Austrian birch point cloud. **(A)** Normalized cumulative number of voxels with at least N points calculated for the Finnish birch crown point clouds and for the full Austrian birch point clouds. **(B)** Normalized cumulative number of voxels with at least N points calculated for the selected branch point clouds differentiated from the full Austrian point clouds.

The question arises, which density of the point cloud is required for doing the first or the second type of movement analysis, i.e., height percentiles vs. points on individual branches. To achieve this, the point density must be quantified. This was done by calculating the number of populated 5 × 5 × 5 cm^3^ voxels for the point clouds, and this was further monitored through time. The normalized cumulative number of voxels populated with at least a given number of points is illustrated in Figure 6 for both cases. In other words, the metric shows how many voxels contain at least n points within the voxel point cloud. This value is normalized by dividing through the number of voxels which have at least 1 point. The point density graphs were drawn at three different times in order to evaluate whether scanning time would have had a significant effect on point cloud density due to any external factors.

In the Finnish full canopy point clouds, the Figure 5A illustrates clearly how the cumulative point density in voxels decreases rapidly. This means that most of the populated voxels had only a few hits in them. Only about 35–40% of the populated voxels have five or more laser returns localized in them. There is also about a 5% percentage point difference between the scans taken at sunset and at night. In the Austrian point cloud, the corresponding number shows that over 65% of the populated voxels have at least five hits in them and there is no significant difference between the scans taken at different times.

The amount of voxels with one or two laser returns is important as they represent spatially isolated returns of which a significant portion may consist of noise, and partial or otherwise low quality hits on target. The graphs in Figure 6A) show that while there is some variation in the point density on the crown and tree level, the point densities between different scans drop in a consistent manner for both scanners regardless of scanning time.

The graphs in Figure 6A) showed the point density for the full crown and tree point clouds. Thus, the inherently noisy backsides of the point clouds with lower point densities are also included. Therefore, similar graphs were also produced separately for the differentiated Austrian birch branches (Figure 6B) that were used for movement measurements to see if they would show different point density variations compared to the full tree point cloud. The branches were selected on the outer edge of the birch and with clear visibility to the scanner (Figure 2). The graphs show that about 85% of all populated voxels inside the bounding boxes of branches 1 and 2 have five or more hits, and over 75% of the voxels have 10 or more hits. The only exception here is the sunrise measurement of the branch 1, where the results are several percentage points lower. Branch 3 results show systematically lower point densities. About 80% of these voxels have at least five hits, and about 65% of the voxels have at least 10 hits. In general, the number of multi-hit branch voxels is over 10% points higher than the number of corresponding voxels in the whole birch point cloud.

Results on the point density variation in the Finnish and Austrian datasets and their effect on manual branch point selection give a rough metric to estimate required point densities for future studies. The representative point monitoring was possible with manual selection for all branches delineated from the Austrian datasets, whereas for the Finnish data similar monitoring was not possible due to lower point density. The lowest tested branch point density here had about 80% of hits coming from voxels with at least five points or more, which would correspond to a point density of at least 40,000 pts/m^3^. Voxels with 10 or more hits (at least 80,000 pts/m^3^) constituted about 65% of all voxels. In the Finnish dataset, the voxel population ratio of over 65% was obtained only when voxels with at least two points (16,000 pts/m^3^) were included. Thus, to perform a similar point selection from a natural birch branch surface (i.e., without additional reflective material), the point densities should be at least close to 40,000 pts/m^3^. This estimated value is not a universal lower limit, but it gives an initial approximation for planning new similar experiments.

Measurement settings and weather conditions were also similar between the experiments:

- Both experiments were carried out with a single scanner setup. Therefore, only one side of the test trees could be monitored over the duration of the experiments. This is sufficient to determine individual branch movements, but determination of the whole crown movement is not possible and would require a minimum of two separate scans from different positions, preferably more. Multiple scans would allow branch movement determination around the tree to get a better insight on possible directional movement differences within the crown. Multiple scans would also provide better information about effects related to growing location or sun position.
- In both experiments the weather was similar, with no observed wind and no rainfall during night. As high-resolution laser scans require typically minute-long collection times for tree-sized objects, this means that the measurements are susceptible to occasional gusts of wind and need to be accounted for in measurement planning. In order to eliminate most wind effects from point clouds, external wind covers would be needed or the scans should be performed in an enclosed setting, for example in a greenhouse. However, in our experiments no disturbance by wind occurred.

## Discussion

### The study in context with previous research

In this study we quantified a diurnal cycle of branch motion in mature birch (*Betula pendula)* trees growing under natural conditions, and therefore demonstrated the potential of TLS point clouds to monitor diurnal branch movements in birch trees. To our knowledge, the study is the first to report overnight branch and crown movement with centimeter level spatial resolution and with (less than) hourly intervals. The study approach presented here is novel in utilizing TLS point clouds with short interval outdoor scanning.

The study comprises two separate measurement settings with different equipment and geographic location, namely Finland and Austria. Target objects were individual birch trees located in the study areas and night time movements were detected by scanning their canopies for a period lasting from sunset to sunrise. The results obtained from the measurements showed that the crown movement in the Finnish and the branch movement in the Austrian study case presented similar temporal response. Close to sunrise, the branches were hanging lower than at the time of sunset. Detected crown and branch movement amplitudes varied from a few centimeters up to 10 cm from their initial locations at sunset, depending on the position of the branch and the measurement point on it. The movements were observed to happen systematically over a time span of several hours, which ruled out occasional wind effects.

Both the molecular background of the circadian rhythm and the resulting movement of various plant parts (the leaf, stem, and flower) have been extensively described for small herbs growing under laboratory conditions. The circadian activity pattern of trees is also of interest, both for generalizing the findings of experimental chronobiology and for commercial use of tree products such as the tree sap [e.g., for the gum tree (*Hevea brasiliensis*)] and the wood (which is best harvested when it has a low water content). Understanding ecophysiological processes of individual trees, including their diurnal water use pattern and how this changes under water stress is becoming increasingly important for climate research, as near-global coverage of high-resolution remote sensing has revolutionized the up-scaling of findings from individual tree-based models to continental scale (Shugart et al., 2015). Until now, *in situ* measurements at the scale of full trees were not possible due to the lack of a non-invasive, non-contact method with high spatial accuracy. Our study demonstrates that TLS satisfies these criteria.

To our knowledge, previous and present TLS time series literature on vegetation mainly concentrates on detecting seasonal changes. The seasonal change studies have mainly focused on collecting physiological parameters, like leaf or needle chlorophyll content (Hakala et al., 2014; Nevalainen et al., 2014), or to follow the growth and phenological changes by studying changes in Leaf area index (LAI), plant area index (PAI), and Plant Area Volume Density (PAVD), e.g., in Griebel et al. (2015) and Calders et al. (2015). A study by Hosoi and Omasa (2009) determined the seasonal changes in vertical leaf area density (LAD) profiles. Measurement intervals in these studies vary from daily and weekly measurements to individual seasonal scans. These scan intervals are sufficient for detecting overall changes on crown level, but cannot capture systematic inter—and intraday dynamics as reported here.

Most of the TLS time series studies are performed during light hours, mainly due to technical restrictions that require presence of measurement staff to set up and monitor the data collection. A change to this is a new operational system, VEGNET, that has been developed and successfully applied for long-term forest monitoring (Portillo-Quintero et al., 2014; Griebel et al., 2015). The main limitation of VEGNET is its limited spatial resolution and a fixed angle rotation plane that have been designed to monitor overall crown structural dynamics around the system instead of focusing on individual trees. The VEGNET operates night time to optimize its ranging capabilities and to minimize possible wind effects. In general, any wind or local airflows present a significant source of noise in TLS point clouds and have to be accounted for either during the measurement. Another reported error source is precipitation on the scanning equipment and on target.

Another feasible approach to collect longer-term time series data with high temporal resolution is short-interval photography. Li et al. (2013) performed a plant growth analysis study where they studied the structural changes in a pot plant in laboratory conditions for 35 days where the same viewing geometry was repeated every 5 min. A 4D (3D structure and time) point cloud representation was then post processed from the imagery to study geometrical changes in the plant. Nijland et al. (2014) used infrared converted consumer grade cameras to collect plant health and phenology information in an outdoor setting for several months with 1-h interval. The main weakness of the imaging approach is the requirement of external lighting and comprehensive radiometric calibration to guarantee correct radiometry between imagery taken in dynamic lighting conditions. Li et al. (2013) applied constant lighting on their targets, whereas Nijland et al. (2014) had to limit their outdoor imaging sequences to light hours. Neither solution allows studying of possible dark time dynamics in plants as reported in this study.

## Summary and future work

The study scope was limited to analysis and quantification of the geometric movements in birch crown and branches. The validation of possible mechanisms behind the movement was not possible within the study scope and further investigations are required. Possible mechanisms contributing to the branch and crown movement may be related to plant water balance or to plant photoperiodism, but their validation was out of the scope of this article.

Circadian changes in plants have been studied extensively in plant physiology and they can be quantified with high detail in laboratory conditions or *in-situ* for individual plants and their parts (e.g., Perämäki et al., 2001). However, extending the results to larger areas is prohibitively laborious. We conclude that whereas laser scanning point clouds are not able to give as detailed information about the physiological changes in plants as laboratory or on-the-spot measurements, they still have a significant potential to provide additional geometrical information that can be correlated with the physiological measurements, and then possibly extended to cover whole plants in their natural environment and over wider areas. Perhaps the most important open question is whether the observed branch movements take place under the influence of light from sunset and sunrise, or if they are independent from light and governed by the internal circadian clock of the plant. The fact that some branches started returning to their daytime position already before sunrise would suggest this latter hypothesis, but the temporal frequency of our investigations is not sufficient for confirming or rejecting it: ideally, measuring several full 24-h cycles of a tree isolated from natural light would be required for this.

To explore the potential of TLS-based sleep movement of trees as an indicator of water use and water stress, further studies are required with simultaneous physiological measurements of trunk diameter, water potential, and photosynthetic activity and reference comparisons. This will allow (i) quantification of the limits of reliability of different terrestrial laser scanners to detect the temporal movements in different plant and tree species and (ii) modeling and further simulation of the relationship between the detected geometric behavior and direct physiological observations of plant water use and circadian rhythms.

## Author contributions

EP is the main author and took part in the planning of both experiments, collection of Finnish data, and performed the data analysis. CB, GM, and NP planned the Austrian dataset collection and contributed to the writing. MP planned and arranged the collection of Austrian dataset and contributed to writing the manuscript. MW processed the Austrian dataset for analysis. AZ strengthened introduction and discussion from biological and ecological perspective, and commented the whole manuscript.

## Funding

This article received support from Academy of Finland grants no. 265949 and no. 272195, and from the Finnish foundations mobility grant “Tutkijat maailmalle, osaamista Suomeen.” AZ was supported by the OTKA grant PD 115833 of the Hungarian Research Fund.

### Conflict of interest statement

The authors declare that the research was conducted in the absence of any commercial or financial relationships that could be construed as a potential conflict of interest.

## Supplementary material

Video 1 **Animation of the overnight movement in the Finnish birch point cloud from sunset to sunrise**. Video is created by drawing birch point cloud of each scan collected during night. The point cloud scanned around sunset (19:40 o'clock) is drawn with black as a reference. The laser scanner was located in origin (0,0).
